# Categorising trajectories and individual item changes of the North Star Ambulatory Assessment in patients with Duchenne muscular dystrophy

**DOI:** 10.1371/journal.pone.0221097

**Published:** 2019-09-03

**Authors:** Francesco Muntoni, Joana Domingos, Adnan Y. Manzur, Anna Mayhew, Michela Guglieri, The UK NorthStar Network, Gautam Sajeev, James Signorovitch, Susan J. Ward

**Affiliations:** 1 Dubowitz Neuromuscular Centre, UCL Great Ormond Street Institute of Child Health & Great Ormond Street Hospital, London, United Kingdom; 2 National Institute for Health Research Great Ormond Street Hospital Biomedical Research Centre, UCL Great Ormond Street Institute of Child Health, London, United Kingdom; 3 John Walton Muscular Dystrophy Research Centre, Newcastle University, Newcastle, United Kingdom; 4 Collaborative Trajectory Analysis Project, Cambridge, Massachusetts, United States of America; 5 Analysis Group Inc., Boston, Massachusetts, United States of America; University of Otago Division of Health Sciences, NEW ZEALAND

## Abstract

Functional variability among boys with Duchenne muscular dystrophy (DMD) is well recognised and complicates interpretation of clinical studies. We hypothesised that boys with DMD could be clustered into groups sharing similar trajectories of ambulatory function over time, as measured by the North Star Ambulatory Assessment (NSAA) total score. We also explored associations with other variables such as age, functional abilities, and genotype. Using the NorthStar Clinical Network database, 395 patients with >1 NSAA assessment were identified. We utilised latent class trajectory analysis of longitudinal NSAA scores, which produced evidence for at least four clusters of boys sharing similar trajectories versus age in decreasing order of clinical severity: 25% of the boys were in cluster 1 (NSAA falling to ≤ 5 at age ~10y), 35% were in cluster 2 (NSAA ≤ 5 ~12y), 21% in were cluster 3 (NSAA≤ 5 ~14y), and 19% in cluster 4 (NSAA > 5 up to 15y). Mean ages at diagnosis of DMD were similar across clusters (4.2, 3.9, 4.3, and 4.8y, respectively). However, at the first NSAA assessment, a significant (p<0.05) association was observed between earlier declining clusters and younger age, worse NSAA, slower rise from supine, slower 10 metre walk/run times, and younger age of steroid initiation. In order to assess the probability of observing complete loss of function for individual NSAA items, we examined the proportion of patients who shifted from a score of 1 or 2 at baseline to a score of 0. We also assessed the probability of gain of function using the inverse assessment and stratified the probability of deterioration, improvement–or static behavior–by age ranges and using baseline functional status. Using this tool, our study provides a comprehensive assessment of the NSAA in a large population of patients with DMD and, for the first time, describes discrete clusters of disease progression; this will be invaluable for future DMD clinical trial design and interpretation of findings.

## Introduction

Duchenne muscular dystrophy (DMD) is a recessive, X-linked neuromuscular disorder caused by mutations in the dystrophin gene, and is characterised by progressive degeneration of skeletal and cardiac muscles [1, 2]. DMD predominantly affects males, with the prevalence in different countries estimated between 17.2 to 69.3 x10^-6^ [1] and a pooled worldwide prevalence of 4.78 per 100,000 (95% confidence interval: 1.9–11.8) [1, 3].

The primary manifestation of patients with DMD is progressive muscle weakness, which impairs walking, motor function, posture, breathing, and leads to a progressive decline in cardiac function [4]. Symptom onset is typically between the ages of 3 and 5 years [5]. Although ambulatory function may initially improve in younger patients due to growth and development, the progression of muscle pathology leads to inexorable progression of weakness leading to loss of ambulation by the age of 13 years [6, 7]. There is no cure for DMD, and the goal of treatment is to manage symptoms, slow disease progression, and delay disability. Glucocorticoid therapy, which has been shown to improve motor and pulmonary functions, and potentially delay cardiomyopathy, is currently part of the standard of care in patients with DMD [8–11].

In a recent nationwide natural history study in the United Kingdom (UK), the median age of loss of ambulation was shown to be 12 years for boys on intermittent glucocorticoid regimens (i.e., 10 days on, 10 days off), and 14.5 years for boys receiving daily glucocorticoid (p = 0.13) [12]. In two other studies performed in the United States (US), the clear effect of chronic glucocorticoids in slowing down time to all disease progression milestone events was evident for patients treated for 1 year or longer, compared to patients treated for less than 1 month or never treated [13, 14]. These two studies clearly highlighted the reduced risk of losing clinically meaningful mobility and upper limb function across the lifespan as well as reduced risk of death for patients with DMD treated with glucocorticoids.

More recently, a number of therapies, which address specific mutations, have been approved in the US (eteplirsen, a drug that induces skipping of exon 51 can be beneficial for ~15% of all boys affected with DMD as they carry deletions amenable to exon 51 skipping [15, 16]) or in Europe (ataluren, inducing read-through nonsense mutations, which occur in ~10–15% of all boys with DMD[17]), and are now commercially available in the respective countries.

The North Star Ambulatory Assessment (NSAA), a validated functional rating scale specifically developed to measure ambulatory performance in boys with DMD [18–20], is a widely-used measure of motor ability used both in natural history studies and as an outcome measure in clinical trials. The NSAA scale is composed of 17 items, and was developed to evaluate changes in gross motor ability (i.e., ability to rise from the floor, move from sitting to standing, jump, run, and ascend/descend steps) [18, 21]. Scores range from 0–34 (0–100 for the linearised version [22]), with higher scores indicating better motor function. Each item is scored between 0 and 2 (0 –unable to perform independently; 1 –able to perform with assistance; 2 –normal, able to perform without assistance). The NSAA has been shown to be reliable in a multicentric setting [19] and demonstrates clinical validity. The total score has also shown significant correlation with quality of life, especially with physical function components [23]; however, that study also highlighted the complex relationship between functional changes and items captured by the PedsQL quality of life questionnaires.

The NSAA score declines on average 8 units/year after the age of 7 years on the linearised scale and 4 points in the raw score [24]. However, a wide clinical variability is recognised among patients with DMD. Therefore, there have been several studies analysing the impact of factors that could contribute to this variability, including different glucocorticoid regimens and patient specific DMD genotypes. Indeed, several studies demonstrated that deletions amenable to exons 44 and 46 skipping are associated with decline at a slower rate over 2 years while 53 and 51 skippable deletions are associated with a faster decline [25–27].

Recent studies have also estimated the NSAA Minimally Clinically Important Difference using a distribution-based method applied to the linearised scale and applied to boys affected by DMD receiving different glucocorticoid regimens [22].

In recent longitudinal studies, the NSAA has been used in combination with other outcome measures to assess decline in motor ability [17, 24, 28]. Among these, a prospective 10-centre study of boys with DMD reviewed scores on NSAA, timed 10 metre walk/run test, timed rise from floor, and six-minute walk test (6MWT) distances [21], and noted that the NSAA total score reliably declined with age, correlated with the other measures, and that patients treated with daily glucocorticoids had better scores overall. Correlations showed that a combination of these outcomes captured decline effectively. Another study that charted the natural history of patients with DMD over 3 years using the NSAA and 6MWT also noted a sustained overall decline in NSAA scores over time, and that boys not on glucocorticoid treatment had a steeper decline [29]. Both studies observed good agreement between the NSAA and 6MWT [21, 29]. A previous study conducted by the Collaborative Trajectory Analysis Project performed a cluster analysis for the 6MWT and was able to identify clusters of boys with DMD with different trajectories of ambulatory function [30] such that the majority of the variation in 6MWT outcomes occurred between rather than within these separate clusters.

To date, a comprehensive cluster analysis has not been performed for the NSAA, and this is now particularly relevant as the NSAA is being used as an outcome measure in clinical trials for patients with DMD (such as the phase III trial of ataluren [31]; trials testing antisense oligonucleotides therapies—SRP-4045 [32], SRP-4053 [33] and anti-myostatin therapy studies [34, 35]). In a recently published phase III trial on ataluren, exploratory analyses of NSAA items showed promising signals for drug efficacy [17, 36], and have contributed to growing interest in the NSAA items as a source of outcome metrics for drug efficacy.

A better understanding of the varied nature of natural history of NSAA scores in patients with DMD can aid in the assessment of treatment efficacy, in the design of clinical trials including defining better inclusion and exclusion criteria and importantly in the identification of variable disease progression rates [24]. Therefore, the aims of this present study were to: (1) assess whether patients with DMD could be clustered into groups sharing similar trajectories of ambulatory function over time as measured by the total NSAA score, (2) to describe the pattern in which skills are lost or gained at different ages in patients with DMD, and (3) assess the effect that different genotypes and glucocorticoid therapies have on the longitudinal progression of disease in patients with DMD. We used latent class trajectory analysis of longitudinal NSAA scores to identify clusters of patients with similar trajectories; a similar analysis has previously identified different disease trajectories and better characterisation of natural histories of cardiovascular diseases and mental health conditions [37, 38], and to identify associations between patient outcomes and biomarkers [39] or interpret treatment effects in clinical trials [40–43]. The pattern of gain and loss of skills, together with the timing and significance of these changes in NSAA items, was assessed. We also quantified probabilities of change in individual items (improvement or deterioration) for the total population (shift analysis), and further refined these findings through stratification of patients by age or by functional status in timed tests.

## Study design and methods

### Patient selection and outcomes measurement

The NorthStar Clinical Network and database includes information on regular assessments of patients with DMD from 24 paediatric specialist neuromuscular centres regularly followed in the UK (http://www.northstardmd.com/about.html).

Functional assessments were conducted by physiotherapists at each of the centres. A training program and standard operating procedures existed across all the participating sites to ensure standardisation of procedures. Clinical and physiotherapy assessments were recorded in forms specifically developed for that purpose and centrally collected for analysis.

The project followed Caldicott Guardian regulations and information was entered in the database after written informed consent was obtained from patients' parents. Only anonymous, de-identified data were analysed. All clinical investigations were conducted according to the principles expressed in the Declaration of Helsinki, following Caldicott Guardian approval.

For this study, patients with a diagnosis of DMD, with more than one NSAA assessment score and recorded dystrophin genotype were included. The functional assessments spanned the years 2004 to 2015 and included NSAA, timed rise from supine, and timed 10 metre walk/run. Information regarding ages at glucocorticoid initiation, glucocorticoid type and dosing were also captured in the database and were used in the analysis. None of the patients in this study were in clinical trials of an experimental therapy.

### Statistical analysis

Patient characteristics at the first NSAA assessment were summarised using means and standard deviations for continuous characteristics and counts and proportions for categorical characteristics.

Longitudinal trajectories of the NSAA total score versus age were studied using latent class trajectory analysis. These analyses assessed whether the observed trajectories of NSAA versus age were adequately described by a single underlying mean trajectory or, alternatively, by a mixture of two or more underlying mean trajectories, with each mean trajectory corresponding to a latent class [44–46]. Within each latent class, the mean NSAA total score was modeled as a quadratic function of age, thus allowing for periods of increasing and decreasing NSAA total score. Models with one, two, three, etc., latent trajectory classes were fit using maximum likelihood. Sensitivity analyses considered more flexible functions of age (i.e., including cubic terms) and autoregressive error models. Model fits were evaluated using Akaike's Information Criterion (AIC) and the Bayesian Information Criterion (BIC), for which lower values indicated a more favorable balance between model fit and model complexity.

To form clusters, patients were assigned to the class for which they had the maximum posterior probability of membership, i.e., the class to which they were most likely to belong to based on the model fitted. Confidence in these assignments was assessed by averaging the assignment probabilities across patients within each class. Proportions of patients with function on individual NSAA items (i.e., score > 0), and average trajectories for the timed rise from supine and the timed 10 metre walk/run, were estimated for each latent class. All analyses were implemented using the lcmm package in R [46].

Following the recently described shift analysis method, where the complete loss of ability was examined and compared to the partial loss of an ability [17], we performed an analysis of the loss of each of the individual functions on the NSAA by examining the proportion of patients who shifted from a higher to a lower NSAA item score between two NSAA assessments separated by ≤ 16 months. For each NSAA item, each such follow-up interval was classified into one of the three following groups based on a change in that item from the beginning to the end of the interval:

- Shift up (improved function): item score increases from 0→(1,2) or 1→2
- Shift down (worsening function): item score decreases from 2→(0,1) or 1→0
- No change: item score stays the same, i.e., 0→→0 or 1→1 or 2→2

For each of these analyses, we subdivided the patients with DMD into three age range categories based on their age at baseline (<7, 7–12, and >12 years old) and into functional status cohorts based on 10 metre walk/run (<6s, 6-10s, > 10s) or rise from supine (<4s, 4-8s, >8s). Rates of shift were calculated on an annualised basis using Poisson regression with offset terms to account for the duration of follow-up between assessments. All pairs of NSAA assessments separated ≤ 16 months were included in the analysis. Generalised estimating equations were used to account for correlation across multiple intervals from the same patient [47].

## Results

### Patient characteristics

An ‘n’ of 395 patients with DMD were included in the study, with ages ranging from 1.8 to 16.7 years at first assessment; average ± standard deviation of age at first assessment was 7.1 ± 2.6 years, average age at diagnosis of DMD was 4.1 ± 2.2 years and average NSAA total score at first assessment was 21.8 ± 7.1 (Table 1). Among these patients, a total of 2,144 NSAA assessments were available for analysis. Across this assessment of NSAA, the mean age was 8.6 ± 2.7 years. The duration of follow-up ranged from less than 1 year to 9.3 years, with a median of 2.8 years across patients. The median time between assessments was 7 months, and 85% of assessments occurred within one year of the most recent prior assessment.

**Table 1:** Patient characteristics at first NSAA assessment.

	← Faster progression			Slower progression →		
Patient characteristics	All patients (n = 395)	Class 1 (n = 105)	Class 2 (n = 135)	Class 3 (n = 80)	Class 4 (n = 75)	P-value^a^
**Demographics**	**Mean +/- s.e. **	**Mean +/- s.e.**	**Mean +/- s.e.**	**Mean +/- s.e.**	**Mean +/- s.e.**	
Age (years)	7.1 ± 2.6	6.8 ± 2.5	6.4 ± 1.9	7.3 ± 2.8	8.3 ± 3.3	< 0.001
Age range (years)	1.8–16.7	3.3–14.8	2.3–10.7	1.8–13.7	2.6–16.7	
Age at diagnosis of DMD (years)	4.1 ± 2.2	4.1 ± 2.2	3.8 ± 1.8	4.2 ± 2.4	4.7 ± 2.4	0.10
**Glucocorticoid use**^**b**^	**n (%) **	**n (%) **	**n (%) **	**n (%) **	**n (%) **	
Use recorded at any time	348 (88.1)	89 (84.76)	119 (88.15)	74 (92.5)	66 (88)	0.46
Glucocorticoid naïve at first assessment	127 (36.49)	37 (41.57)	46 (38.66)	26 (35.14)	18 (27.27)	0.2944
Age at initiation, years	6.2 ± 1.8	6.1 ± 1.6	5.8 ± 1.2	6.4 ± 1.9	6.6 ± 2.5	< 0.01
Glucocorticoid type^b^	n (%)	n (%)	n (%)	n (%)	n (%)	0.56^c^
Deflazacort	12 (3.0)	3 (2.9)	2 (1.5)	3 (3.8)	4 (5.3)	
Prednisone	224 (56.7)	54 (51.4)	78 (57.8)	49 (61.3)	43 (57.3)	
Missing glucocorticoid type	159 (40.3)	48 (45.7)	55 (40.7)	28 (35.0)	28 (37.3)	
**Ambulatory function**^**d**^	**Mean +/- s.e. **	**Mean +/- s.e.**	**Mean +/- s.e.**	**Mean +/- s.e.**	**Mean +/- s.e.**	
NSAA total score	21.8 ± 7.1	17.6 ± 7	22.1 ± 5.8	22.4 ± 7.9	26.3 ± 5.5	< 0.001
Linearised NSAA score	59.7 ± 15.9	50.6 ± 14.6	59.9 ± 12.4	62.2 ± 18.1	69.3 ± 13.9	<0.001
Timed rise from supine (seconds)	5.8 ± 4.6	7.7 ± 5.6	6.1 ± 5.1	5.1 ± 3	3.9 ± 1.8	< 0.001
Timed 10 metre walk/run (seconds)	7.1 ± 3.2	8.1 ± 2.9	6.9 ± 2.5	7.2 ± 4	6.2 ± 3.8	< 0.01
**Dystrophin genotype**	**n (%)**	**n (%)**	**n (%)**	**n (%)**	**n (%)**	
Skip 44	24 (6.1)	6 (5.7)	6 (4.4)	8 (10)	4 (5.3)	0.41
Skip 45	32 (8.1)	12 (11.4)	14 (10.4)	4 (5)	2 (2.7)	0.09
Skip 51	53 (13.4)	16 (15.2)	19 (14.1)	9 (11.3)	9 (12)	0.85
Skip 53	29 (7.3)	4 (3.8)	10 (7.4)	8 (10)	7 (9.3)	0.36
Nonsense	10 (2.5)	2 (1.9)	4 (3)	2 (2.5)	2 (2.7)	0.96

Means ± standard deviations are shown for demographics, glucocorticoid use and ambulatory function; for dystrophin genotype, number of patients are shown (parentheses reflect percentage within a cluster class)

DMD, Duchenne muscular dystrophy; n, number; NSAA, North Star Ambulatory Assessment; s.e., standard error.

^a^ Global test of differences across classes.

^b^ Summarised among patients with steroid use recorded at any time.

^c^ Test of difference in steroid types (prednisone, deflazacort, or missing) across groups.

^d^ Summarised among patients with non-missing data.

**Fig 1:**
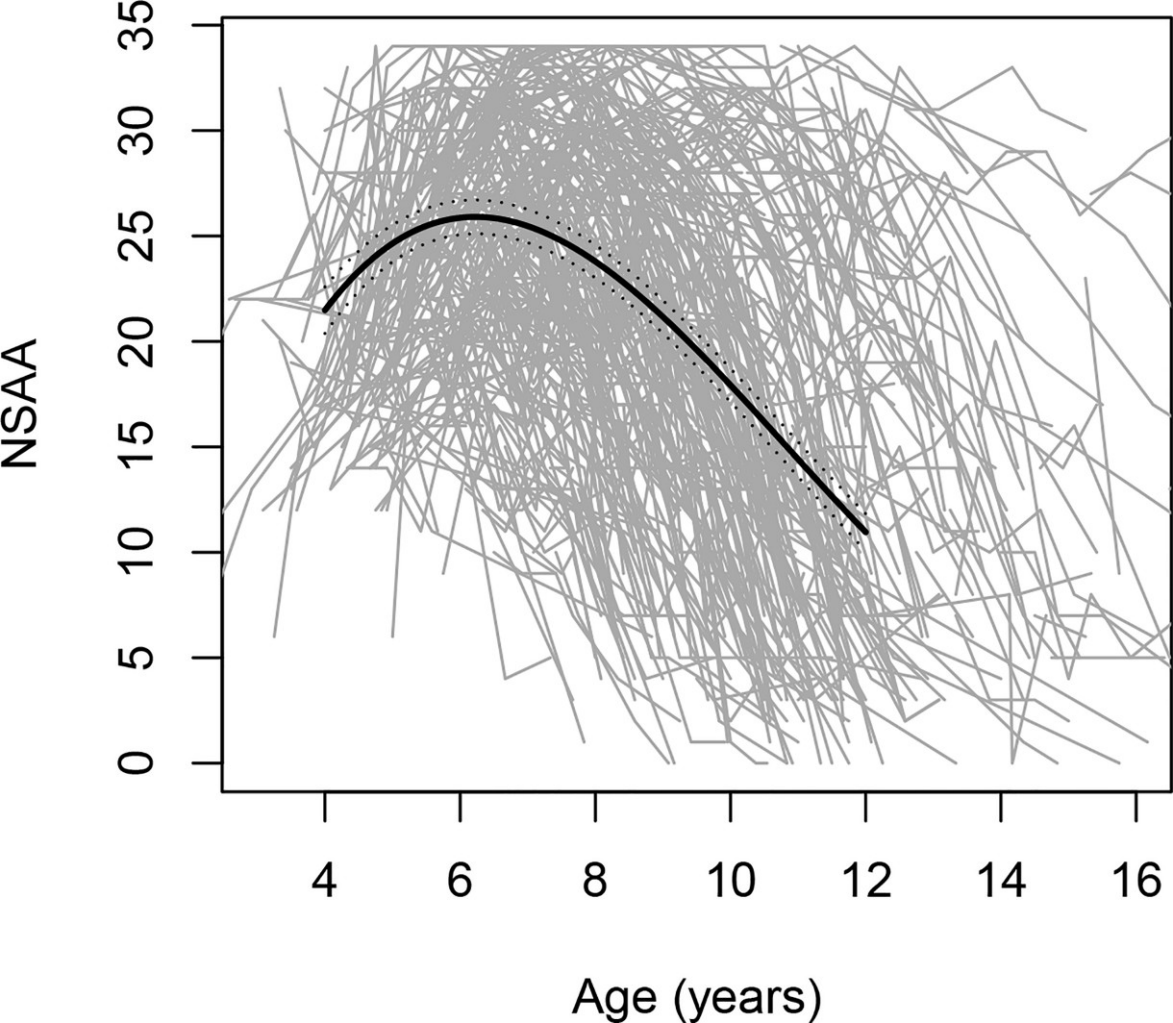
**NSAA total score trajectories for individual patients by age (in grey) and the fitted mean and 95% confidence interval (in black).** Each grey line represents NSAA total scores from an individual patient plotted versus age; the population mean and its 95% confidence bands are shown in black. NSAA, North Star Ambulatory Assessment.

We first determined the overall mean trajectory of NSAA total scores versus age in the studied population. The mean trajectory of NSAA total score versus age of the patients peaked at age 6.3 years with a mean NSAA score of 26; the population mean NSAA total score initially increased at a rate of approximately 3 units per year and, following the peak, eventually approached a rate of decline of approximately 3 units per year (Fig 1).

**Fig 2:**
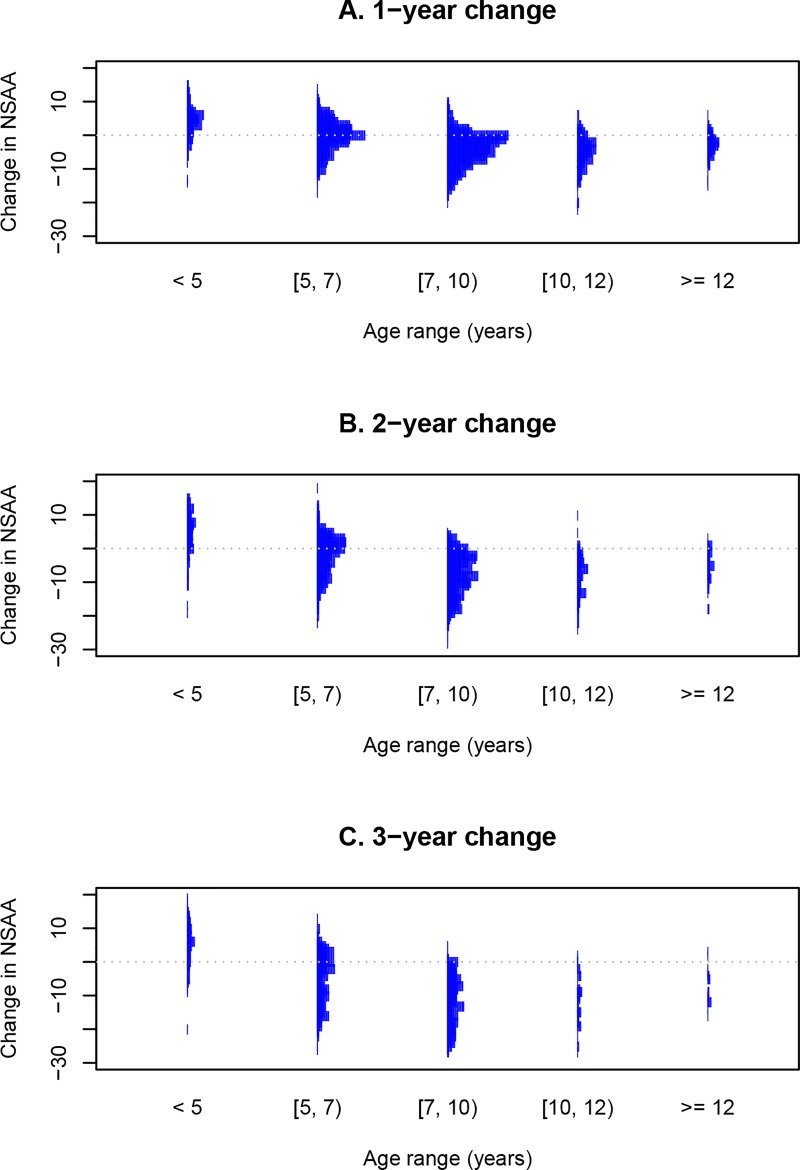
**Histograms of changes in NSAA total scores over (A) 1 year, (B) 2 years, and (C) 3 years among boys with DMD belonging to different age ranges.** DMD, Duchenne muscular dystrophy; NSAA, North Star Ambulatory Assessment.

We then assessed the yearly changes in the NSAA raw and linearised score for all boys affected by DMD stratified by age at recruitment. Fig 2 illustrates the change in raw NSAA total scores in boys with DMD in the different age ranges after 12 months (Fig 2A), 2 years (Fig 2B), and 3 years (Fig 2C). The changes described using linearised NSAA scores segmented by the same age groups are shown in S1A Fig (12-month changes), S1B Fig (2-year changes), and S1C Fig (3 years changes). The coefficient of correlation between the raw and linearised scores was 0.94 at 1 year, 0.96 at 2 years, and 0.96 at 3 years.

### Progression of disease

#### Heterogeneity

**Fig 3:**
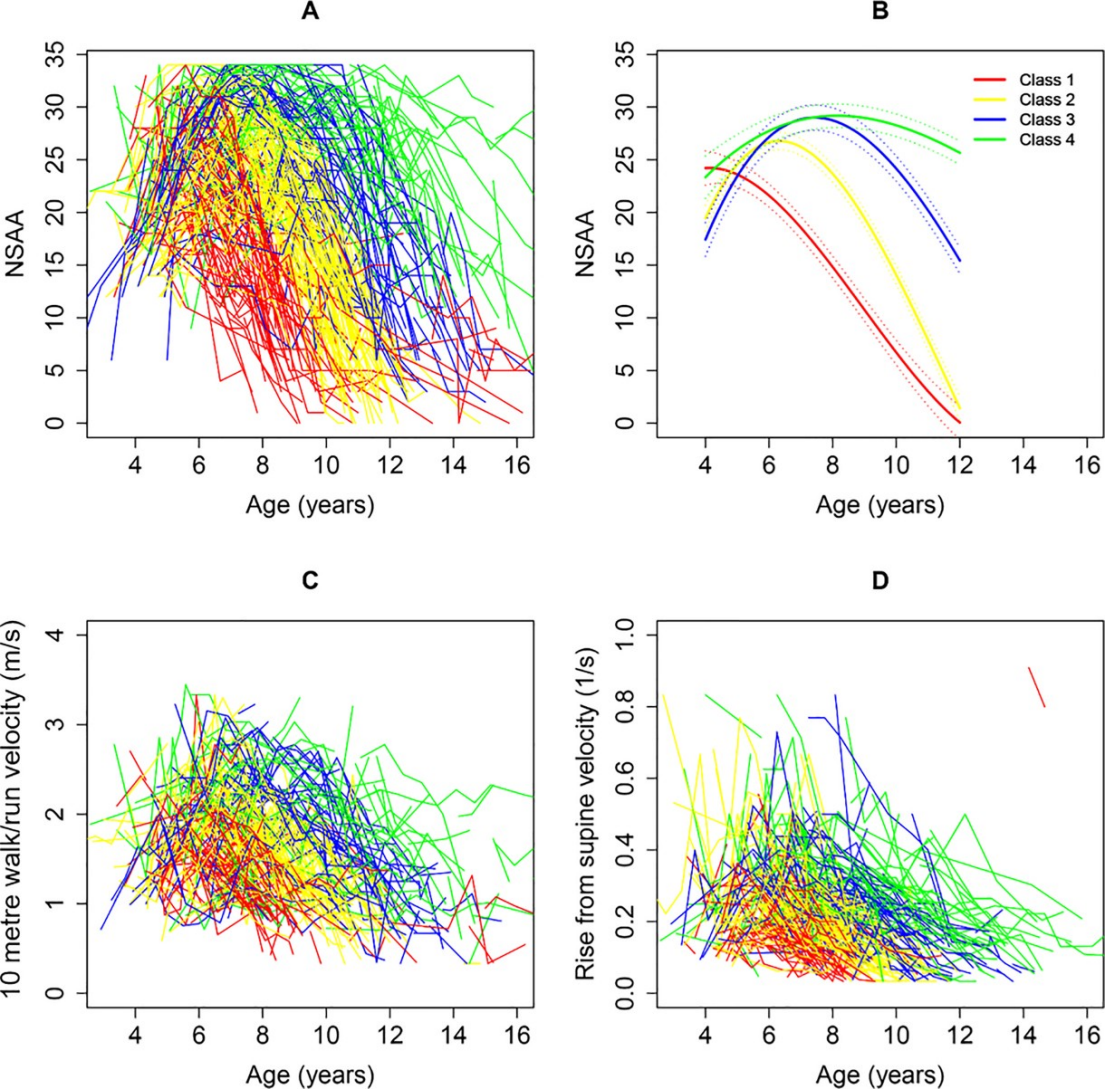
**Individual patient (A) and fitted mean (B) trajectories of NSAA total score by latent class; individual patient trajectories of (C) 10 metre walk/run velocity and (D) velocity of timed rise from supine, all stratified by latent class.** m/s, metres per second; NSAA, North Star Ambulatory Assessment; s, seconds.

In order to assess if disease trajectories could be observed in this patient population using the NSAA, we applied the latent class model with four classes, and this explained the data significantly better than a single mean trajectory (i.e., lower AIC and BIC) (Fig 3A and 3B). We also used models with five and six classes (largest number investigated) which yielded similar findings (data not shown); however, for descriptive purposes, we have used the 4-class model in the discussion. In this 4-class model, confidence in class assignments based on each patient's full history of NSAA total scores exceeded 70% for all classes (S1 Table). Approximately half of the variation in NSAA scores occurred between clusters (R-squared = 0.47). Models with more flexible trajectories did not fit the data substantially better (i.e., did not improve AIC and BIC). Inspection of quantile-quantile plots indicated that residuals were normally distributed, consistent with modeling assumptions.

#### Characteristics

In the model with four classes, patients were distributed based on severity of progression as follows (Fig 3A and 3B): 27% of the patients were in class 1, which showed the fastest progression with most NSAA total scores falling to ≤ 5 around 10 years of age; 34% were in class 2, with NSAA total scores falling to ≤ 5 by approximately 12 years of age; 20% were in class 3, with NSAA total scores falling to ≤ 5 around 14 years of age; 19% were in class 4, which showed the slowest progression, with NSAA total scores remaining > 5 to at least age 15 years. The threshold of NSAA total score < 5, which was selected for this descriptive characterisation, represents the 5^th^ percentile of all recorded NSAA scores and, among patients with a recorded score of < 5 (74 patients) over 90% (68 out of 74) either have a recorded score of 0 (13 out of 74) or a follow-up visit without an NSAA assessment (55 out of 74) within the next year; the remaining 6 had no follow-up assessment in the database.

In addition to the age at which NSAA total score falls below 5, there were other important differences between patients in the four different classes. For example, fewer than 5% of patients with DMD in the fast progressing class (Class 1) achieved an NSAA score above 28; in contrast patients in the slowest progressing class (Class 4) reached a higher score, with ~ 50% of patients achieving a NSAA score above 30. While a smaller portion of patients in the third class also reached a maximum score of 34, these patients began to decline from their peak earlier than boys in class 4 who remained stable for more than 1–2 years longer (Fig 3B). Mean ages at diagnosis of DMD did not differ significantly across the 4 classes, and ranged from 3.8 to 4.7 years across clusters (Table 1). However, the following associations were observed on average for earlier- versus later-declining classes: younger age at first assessment, worse NSAA, slower rise from supine, and slower 10 metre walk/run at first assessment, and younger ages at glucocorticoid initiation (Table 1). In addition, boys in the slower declining classes 3 and 4, who had their first assessments later and started steroids later, were less likely to be steroid naïve at their first assessments than boys with DMD in classes 1 and 2 (on average) (Table 1).

#### Concordance

When patients were grouped by the NSAA-based latent classes, timed 10 metre walk/run (Fig 3C) and the timed rise from supine (Fig 3D) showed patterns of decline across latent cluster classes that were concordant with those of the NSAA total score, with each of these measures entering a period of rapid average decline in the same order across classes. However, performance on the timed rise from supine appeared to decline earlier and more rapidly than on the 10 metre walk/run within each latent class.

#### Associations

Out of the total sample of n = 395 boys with DMD, 347 (or 88%) had a glucocorticoid start age recorded. A total of 224 were recorded to receive prednisone at any time (i.e., including after their first assessment) and a total of 12 were recorded to receive deflazacort at any time; the remainder did not have a glucocorticoid type specified. No significant associations were observed between trajectory classes and type of glucocorticoid received at first assessment (Table 1). Of the 395 patients in this study, 138 patients had a dystrophin mutation amenable to skipping of exons 44, 45, 51, or 53, and 10 patients had nonsense mutations; the remaining 247 patients all had one or more of the many other known mutations that cause DMD [48]. No significant associations were observed between latent classes and type of dystrophin mutation (Table 1).

### Gain, retention, and loss of function on NSAA item skills

**Fig 4:**
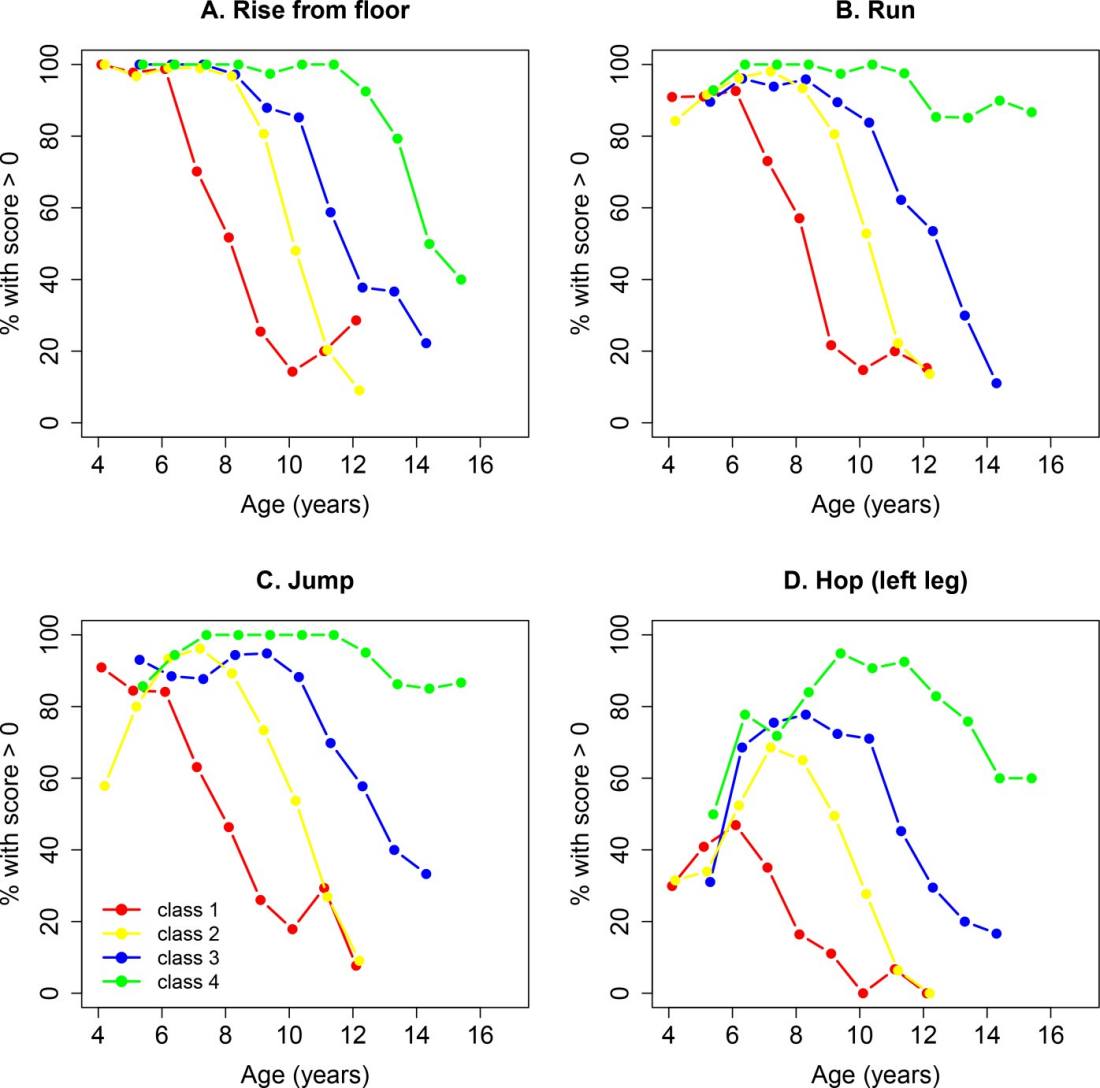
Percentages of patients demonstrating function (NSAA item score > 0) by age and by trajectory class for four of the NSAA items. (A) rise from floor, (B) run, (C) jump, and (D) hop on one leg. NSAA, North Star Ambulatory Assessment.

Individual NSAA item scores showed patterns of decline across classes that were concordant with those of the NSAA total score, with each measure entering the period of rapid average decline in the same order across classes. Fig 4 illustrates representative results on four items.

As illustrated in Fig 4, retention of function to rise from supine, run, jump, and hop each showed patterns of decline across latent cluster classes that were concordant with those of the NSAA total score, with each of these measures entering a period of rapid average decline in the same order across classes with the exception of a patient’s ability to lift their head. Of the various items, hop on one leg showed the strongest maturational effect across the 4 clusters (Fig 4D, hop left leg). Note that less than 50% of the patients in class 1 learn the ability to hop, while more than 90% of boys in the fourth category acquired this skill.

#### Probability of change in NSAA item skills over one year (“shift analysis”)

**Fig 5:**
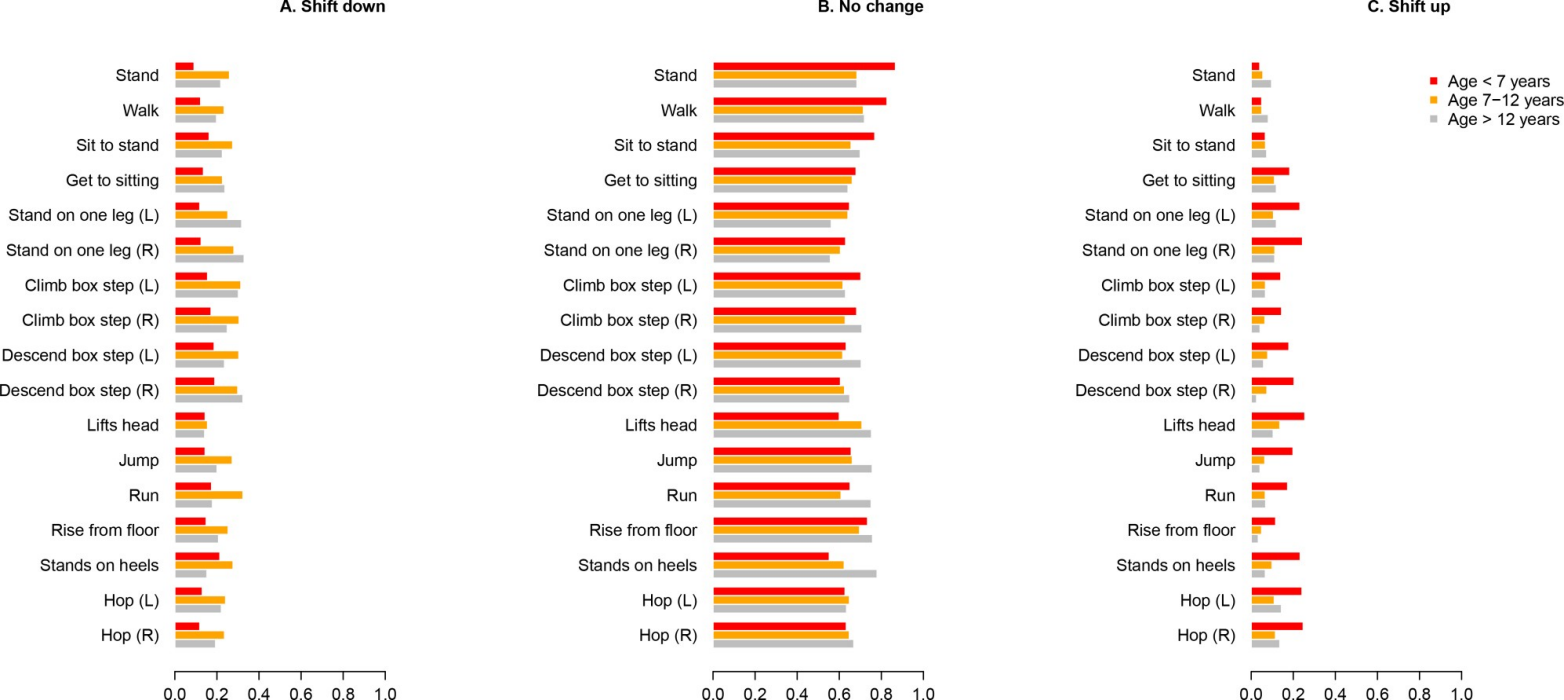
Shifts in NSAA item scores over 1 year: stratification by age. Annualised probabilities of (A) deterioration (shift down); (B) stable function (no change); or (C) improvement (shift up) in the population with DMD stratified by age groups. DMD, Duchenne muscular dystrophy; L, left; NSAA, North Star Ambulatory Assessment; R, right.

Data from 357 patients with DMD who had at least two assessments separated by a maximum of 16 months were included in this analysis. Over the period of approximately one year, the most probable outcome for any given item skill is that function will not change, with patients neither gaining or losing function independent of their age (Fig 5B); the probability of ‘no change’ ranged from 61% (stand on one leg, stand on heels) to 75% (walk, stand).

The probability of shift changes was different for different skills: for example, shifts in lifts head ranged from 15% (shifts up) to 18% (shifts down), whereas shifts in run, and climb or descend box ranged from 10% (shift up) to 26% (shift down) (data not shown).

#### Stratification by age

When analysing the three age range categories, older children (grey bar) were, as expected, at increased risk of shift down, but not all items demonstrated the same degree of risk of loss of function in the different age ranges. Almost invariably the largest differences in the risk of worsening were between the groups with age of <7 years and 7–12 years, with smaller differences between 7–12 and ≥ 12 (Fig 5A). In some cases (for example, walk on heels and running), the risk of worsening was lower in those aged ≥ 12 years than among those aged 7–12 years.

Regarding the shift up (Fig 5C), as expected, the largest probability of improvement was noticed in the children below the age of 7 years (red bars). However, a modest chance of improvement for several items after the age of 7 were evident, notably for hop (14%), standing on one leg (12%), get to sitting (12%), and lifts head (14%). For completion, we also provide a graphical representation of the “no change” probability for the different items in the three age ranges chosen (Fig 5B).

#### Stratification by functional status

**Fig 6:**
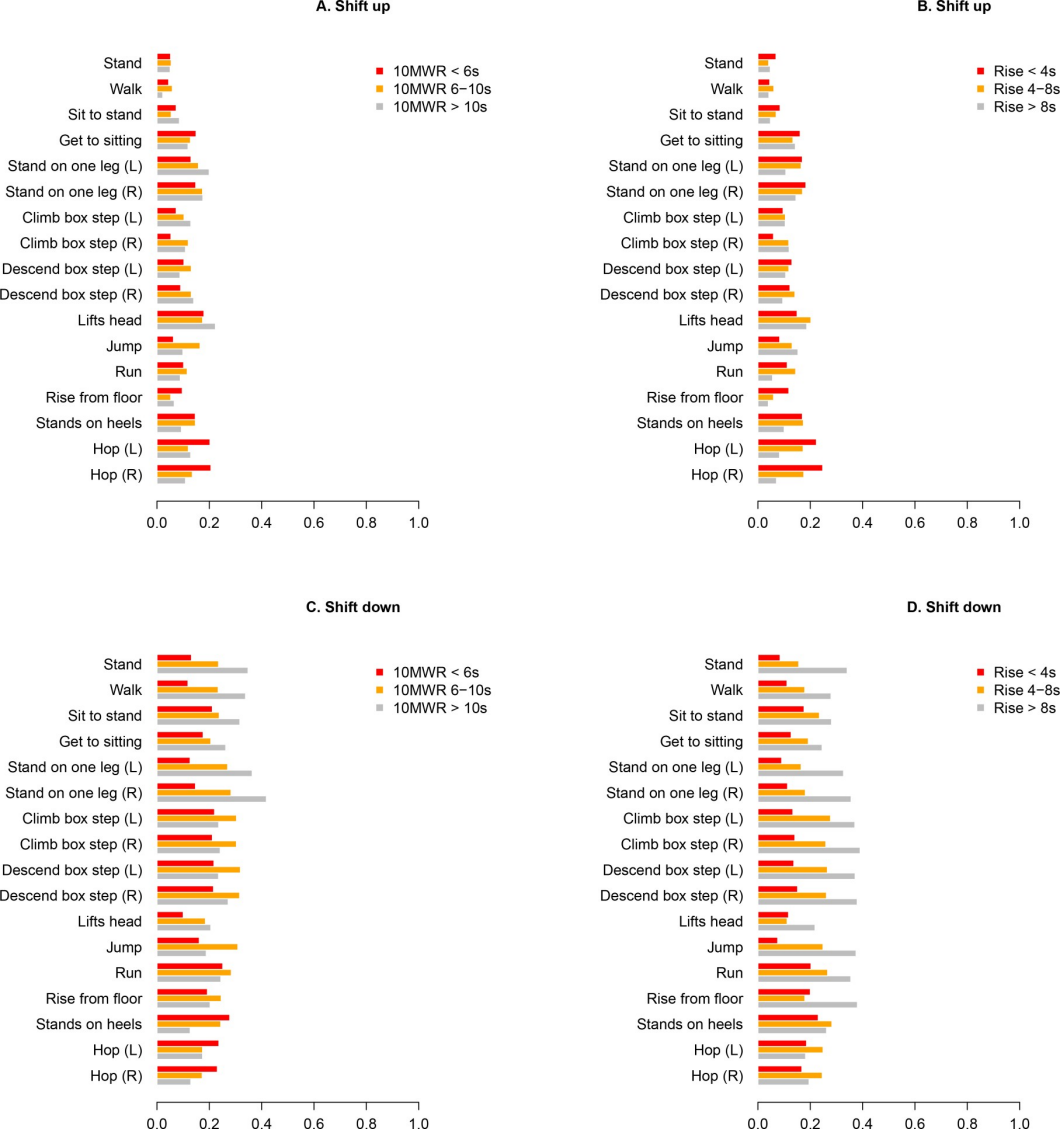
Shifts in NSAA item scores over 1 year: stratification by functional status. Annualised probabilities of shift up in NSAA item scores when stratified by (A) 10 metre walk/run and (B) rise from supine; probability of shift down in (C) 10 metre walk/run and (D) rise from supine. 10MWR, 10 metre walk/run; s, seconds; DMD, Duchenne muscular dystrophy; L, left; NSAA, North Star Ambulatory Assessment; R, right.

Next, we assessed the risk of shift up or down based on baseline function. In particular, we stratified by baseline 10 metre walk/run (with three categories, < 6 seconds; 6–10 seconds; > 10 seconds). Probability of shift up in 1 year in Fig 6A (Probability of Shift down in Fig 6C). We also stratified by baseline rise from the floor (< 4 seconds; 4–8 seconds; > 8 seconds). Probability of shift up in 1 year in Fig 6B (Probability of Shift down in Fig 6D). These cutoffs were chosen based on clinical experience, and are used to facilitate the description of any strong patterns in items scores across groups.

The shift analysis based on functional status captured the different stages of the disease in a more linear fashion compared to the shift analysis by age, i.e., there was less discontinuity between the different categories when shift analysis was performed based on function (Fig 6 versus Fig 5). Overall, the following trends were observed: 1) the pattern of shift up or down was broadly similar for both rise from supine and 10 metre walk/run stratification; 2) patients who completed the 10 metre walk/run or rise from supine rapidly (red bars) were more likely to shift up on hop (20%, 22%) and stand on heels (15%,17%), respectively than on any other NSAA item (Fig 6A and 6B), and had overall roughly similar probabilities of a shift up or a shift down in the following 12 months; and 3) patients with lengthy times to complete 10 metre walk/run or rise from supine were more likely to lose function in the coming year than gain function (grey bars).

## Discussion

In the last decade, there has been increasing effort to develop outcome measures that capture meaningful aspects of the disease course in patients with DMD both in clinics and in prospective natural history studies, and are robust in terms of their construction to enable reliable assessments for clinical trials. Our group has previously described the NSAA, specifically developed for ambulant patients with DMD, as an adaptation of the original Hammersmith Motor Ability Scale [49]. The adaptation was introduced to overcome the ceiling effect of the Hammersmith Motor Ability Scale, with the addition of functions such as hopping and running, typically not observed in glucocorticoid naive children with DMD. In addition, the NSAA also includes items such as the 10 metre timed walk/run test and timed rise from the floor, and underwent Rasch-transformed analysis [22]. The NSAA has since been used as a secondary outcome measure in several trials in which the 6MWT was the primary outcome [16, 17, 50] and is being currently used as a primary or secondary outcome measure in clinical trials. A deeper understanding of the evolution of changes of the individual items with age and disease progression in patients with DMD is therefore an acute need in the scientific community. Previous studies have compared the evolution of changes at one year using both the NSAA and the 6MWT [21], demonstrating a good correlation with the timed rise from the floor and the 6MWT. This and other studies [30, 51] have stressed the high variability in patients’ changes in 6MWT over time, which has complicated the interpretation of results in recently completed clinical trials. In order to address the observed variability, recent studies have grouped boys affected by DMD into classes that shared similar ambulatory function trajectories as measured by the 6MWT, and developed prediction models for 1-year change in the 6MWT among patients with DMD [30, 51]. However, there is no information on how the NSAA could be used to describe the different trajectories of patients with DMD. In addition, while most of the previous studies have described the cumulative population's annual changes and how different glucocorticoid regimens can affect these changes [24], there is little information on how individuals’ item skills are affected both during early stages of DMD, where relative improvement is observed, and during later stages of disease progression. This concept is important as outcome measures such as the 6MWT demonstrate an apparent improvement in distance walked in children of a younger age, before deterioration is observed [48].

Therefore, the main aim of this study was to apply the recent knowledge of the latent classes to the NSAA in a UK population of patients with DMD. Analysis of data from 395 boys with DMD studied in the large ongoing natural history NSAA study in the UK, confirmed the overall tendency of the NSAA scores to decline [12]. As previously demonstrated by Mercuri, et al. who used 6MWT as the outcome, clustering of patients’ longitudinal trajectories of progression on NSAA demonstrated that the data are a better fit to multiple sub-populations of patients with similar trajectories than to a single population [30]. These results should not be interpreted as establishing any specific number of NSAA trajectory clusters in DMD. Rather, there is a continuum of variation across trajectories. The use of four clusters designated in the present study serves as a descriptive tool, and as a reference framework for better characterising changes in ambulatory function across patients.

Up to 27% of boys with DMD belonged to the first class characterised by the fastest progression and with NSAA total score falling to ≤ 5 around 10 years of age; while at the other end of the spectrum 19% of the boys with DMD clustered in class 4, which showed the slowest progression, with NSAA total scores remaining > 5 (indeed > 10) to at least age 15 years. These descriptive analyses help illustrate that different patients with DMD progress through phases of ambulatory decline at different ages, and are qualitatively consistent with the findings from a similar analysis of 6MWT [30]. Like the association with age, the more slowly progressing patients also reached a higher peak total NSAA score. This observation is of note since it is in keeping with the finding that a proportion of patients with DMD over the age of 7 years can still show the possibility of items scores shifting up.

Our study suggests that the younger a patient with DMD is when his ambulatory capability starts declining, the lower his peak NSAA score and the age at which that peak is reached. Patients who show slower progressions reach a higher peak NSAA score, which is reached at a later age. While these observations need to be ratified by further quantitative analysis, they offer the intriguing possibility that there is a meaningful correlation between disease progression in any individual patient with DMD, which is linked to peak function and the age at which it is achieved.

We then analysed if the declines in performance on "timed function tests" occurred with the same time ordering across clusters as declines in the "NSAA total score", and did not find a significant effect of clusters on NSAA item loss. The timed rise from supine and timed 10 metre walk/run also showed patterns of decline across classes that were concordant with those of the NSAA total score.

No significant associations were observed between latent class membership and type of dystrophin mutation, with similar representations of dystrophin genotypes of particular interest (which are potential targets of personalised medicine), being present in all latent classes (Table 1). As an example, patients amenable to skip-51 treatment were the most prevalent in each class (Table 1), as expected from previous studies. Furthermore, the relative proportion of patients with specific genotypes in each cluster class was consistent with the relative distributions described for the broader population with DMD [52].

These observations suggest that dystrophin genotype is not the primary driver of disease progression in patients with DMD. At first glance, these results may seem at odds with previous studies in which small but significant associations have been found between a dystrophin genotype and a functional decline over 2 years [25, 27], as well as the age at which loss of ambulation occurs [12, 24]). Upon closer inspection, it is clear that in any specific cohort–especially in cohorts with a small number of patients–a relatively small imbalance between patients whose disease is rapidly progressing and those whose disease is slowly progressing could translate into apparent differences in functional endpoints that may not reflect the drivers of different trajectories of disease progression [25, 27].

An interesting observation of this natural history study is that while the age at diagnosis of boys with DMD belonging to different cluster classes was similar, boys in faster versus slower progressing clusters tended to have glucocorticoids prescribed earlier. This association is likely to be the result of the more severe rate of progression and functional status impacting the age at glucocorticoid initiation in this group of children, rather than a causal effect of glucocorticoid treatment. Glucocorticoids are indeed recommended at the time of the plateau, before substantial physical decline [11]. In keeping with this interpretation, the mean age at first NSAA visit was higher in boys with DMD belonging to the fourth class.

Understanding predictors of ambulatory trajectories could enable enrichment of clinical trial populations for particular patient trajectories hypothesised to benefit most from treatment during the trial period. It is becoming increasingly clear that a number of genes have a strong effect in modifying disease progression and/or response to glucocorticoid treatment [53–57]; magnetic resonance imaging measures of muscle composition [58–60], or other biomarkers are currently being investigated.

Nevertheless, the assessment of functional abilities in patients with DMD is key for recruitment and evaluations in clinics and in clinical trials. The improved understanding of the different trajectories of the NSAA coupled with the possibility to assign a given patient to a specific cluster is of interest. In clinical trials, this information can be used to select or enrich for a population of patients with more homogeneous trajectories of progression over the duration of the trial, with improved interpretation of the results. From a clinical perspective, understanding a patient’s longitudinal trajectory would enable the medical team and the family to be prepared for an upcoming clinical milestone.

In a recent study, Mc Donald et al. introduced the concept of considering the complete loss of ability to perform each of the 17 individual items of the NSAA score, rather than the total NSAA score [17]. In a post-hoc analysis after 48 weeks, patients given ataluren lost 12.2% (203/1,665) of functions compared with 17.8% (294/1,656) of functions lost by patients given placebo, equating to a 31% reduced risk of loss of function for ataluren-treated patients [17].

To characterise this innovative analytic approach in our large patient population, we assessed the possibility, or risk, of deterioration, of improvement, or of no change for each of the individual items in the total population, as well as across three age range categories. We demonstrated that in the total population, the risk of worsening was not the same for each of the items, with a negligible risk of deteriorating head function; a high risk of worsening in the ability to walk on heels, to climb and descend stairs, to run, hop, and jump. Subdividing the three age range categories showed that there is an increased risk of shift down for the older children, although not all items demonstrated the same degree of risk of loss of function in the different age ranges.

Regarding the shift up, as expected, we documented the largest probability of improvement in younger children. Interestingly, a small but not insignificant chance for improving was also documented after the age of 7 years, particularly for "standing on one leg" and "hop". This observation is consistent with the longitudinal clustering of patients in which more slowly progressing patients (class 3, and particularly class 4) had total NSAA scores that were clearly still improving after the age of 7 years. As the great majority (89%) of our patients were on glucocorticoids, we are not able to demonstrate if this observed improvement, especially in the younger boys, is related to glucocorticoids, which in the UK are introduced at a mean age of 6.4 years [12].

Our data on the probability of shift up, down, or no change has obvious implications for clinical trial design. Most clinical trials in the past decade have deployed inclusion/exclusion criteria based on the age of the patient as a means of excluding patients whose ambulatory function is still improving. However, analyses of both longitudinal trajectories of patients in the NorthStar database, as well as the probability of their item scores shifting up after the age of 7 years, indicates that a meaningful proportion of the patients (~ 40%) are still in a maturation phase where ambulatory function is either improving or likely to be stable over the coming year. Another viewpoint is that, in the case when patients are only segmented using age, one may expect only 25% of patients between 7 and 12 years of age to be in a decline phase as measured by total NSAA score based on our data.

More recently, younger populations of boys with DMD have been recruited into clinical trials (e.g., boys with DMD between 6 and 48 months of age in the "Young DMD study" of eteplirsen [61], or the adeno-associated virus microdystrophin gene therapy trial [62], in which boys with DMD between the age of 3 months to 7 years are recruited). Since DMD is characterised by a progressive loss of muscle mass, trials targeting very young children aim to improve the probability of observing an improvement (versus a stabilisation) in a patient’s function. Current natural history in this younger population, and a clear understanding of the probability of item scores shifting up, is a critical benchmark to help in the interpretation of study outcomes.

Previous studies have demonstrated that functional status at baseline is an important marker of disease progression for the 6MWT [7, 51]. To better understand the relationship of baseline function to probability of NSAA item scores patients were stratified based on function (rise from the floor and 10 metre walk/run test). Our data demonstrates that the shift analysis stratified by starting functional status was a better approach to segment patients into different stages of disease progression compared to the shift analysis stratified by the age of the patient. Once again, this highlights the importance of not considering age in isolation as the main indicator of clinical severity and stage of disease progression in patients with DMD.

The present study has important limitations. This large database describes real-world practice but has some missing data; in particular, NSAA item and total scores may not be measured for patients with poor ambulatory function. Differences in steroid use across clusters represent associations and not causal effects.

## Conclusions

This study characterises the longitudinal heterogeneity of disease progression in children affected by DMD assessed using the NSAA tool. Latent class trajectory analysis identified different classes of progression. Changes in the proportion of patients with functional capability on individual items in the NSAA paralleled latent class cluster membership, and showed that the proportion of patients acquiring the more difficult skills such as hop and jump was more than double in slowly progressing patients versus those whose disease progresses rapidly. Determining the probability of deterioration, and improvement–or static behavior–for each individual item in the NSAA (a shift analysis), which recognises the number of functional activities that will be either completely lost or gained, showed that segmentation by baseline ranges in function resulted in more consistent shifts than segmentation by different age ranges. This information provides novel and useful information for trial design and interpretation of the growing number of clinical trials employing NSAA as the primary outcome measure.

## Supporting information

S1 Table Posterior probabilities of classification based on North Star Ambulatory Assessment versus age. (DOCX)

S1 Fig **Histograms of changes in linearised NSAA scores** over (A) 1 year, (B) 2 years, and (C) 3 years among boys with DMD belonging to different age ranges.
DMD, Duchenne muscular dystrophy; North Star Ambulatory Assessment.
(TIF)

S2 Fig **Fitted mean trajectories** of (A) NSAA total score, (B) 10 metre walk/run completion time and (C) timed rise from supine, all stratified by latent class.
NSAA, North Star Ambulatory Assessment; s, seconds.
(TIF)
