# Supplementary material for: Categorising trajectories and individual item changes of the North Star Ambulatory Assessment in patients with Duchenne muscular dystrophy
Source: PLoS One. 2019 Sep 3;14(9):e0221097. doi: 10.1371/journal.pone.0221097 (PMC6719875; doi:10.1371/journal.pone.0221097)
Supplement: S1 Table — (DOCX) [file pone.0221097.s001.docx]

**S1 Table. Posterior probabilities of classification based on North Star Ambulatory Assessment versus age**

*Numbers on the diagonal (shown in bold) indicate the level of confidence in class assignments relative to a maximum value of 100%. Numbers off the diagonal represent the overlap between classes and the potential for misclassification.*

|  | **Class assignment (most likely class)** | | | |
| --- | --- | --- | --- | --- |
|  | Class 1 | Class 2 | Class 3 | Class 1 |
| **Probability of class assignment** | n=105 | n=135 | n=80 | n=75 |
| Class 1 | **80%** | 12% | 4% | 4% |
| Class 2 | 9% | **71%** | 12% | 7% |
| Class 3 | 4% | 9% | **74%** | 13% |
| Class 4 | 1% | 4% | 16% | **79%** |
|  | 94% | 96% | 106% | 104% |
