# Supplementary figures and images for: Categorising trajectories and individual item changes of the North Star Ambulatory Assessment in patients with Duchenne muscular dystrophy
Source: PLoS One. 2019 Sep 3;14(9):e0221097. doi: 10.1371/journal.pone.0221097 (PMC6719875; doi:10.1371/journal.pone.0221097)

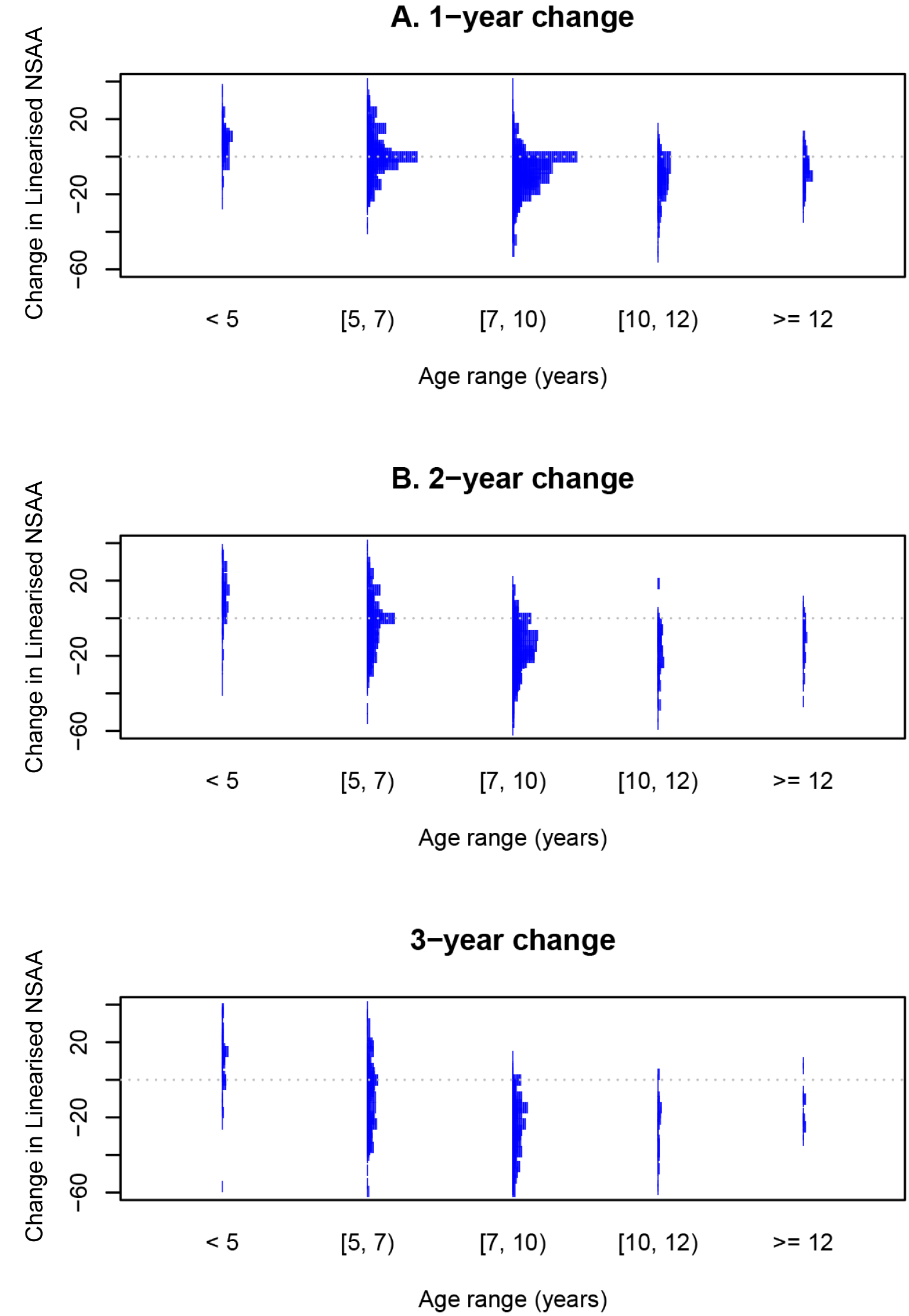

Supplement: S1 Fig — Histograms of changes in linearised NSAA scores over (A) 1 year, (B) 2 years, and (C) 3 years among boys with DMD belonging to different age ranges. DMD, Duchenne muscular dystrophy; North Star Ambulatory Assessment. (TIF) [file pone.0221097.s002.tif]

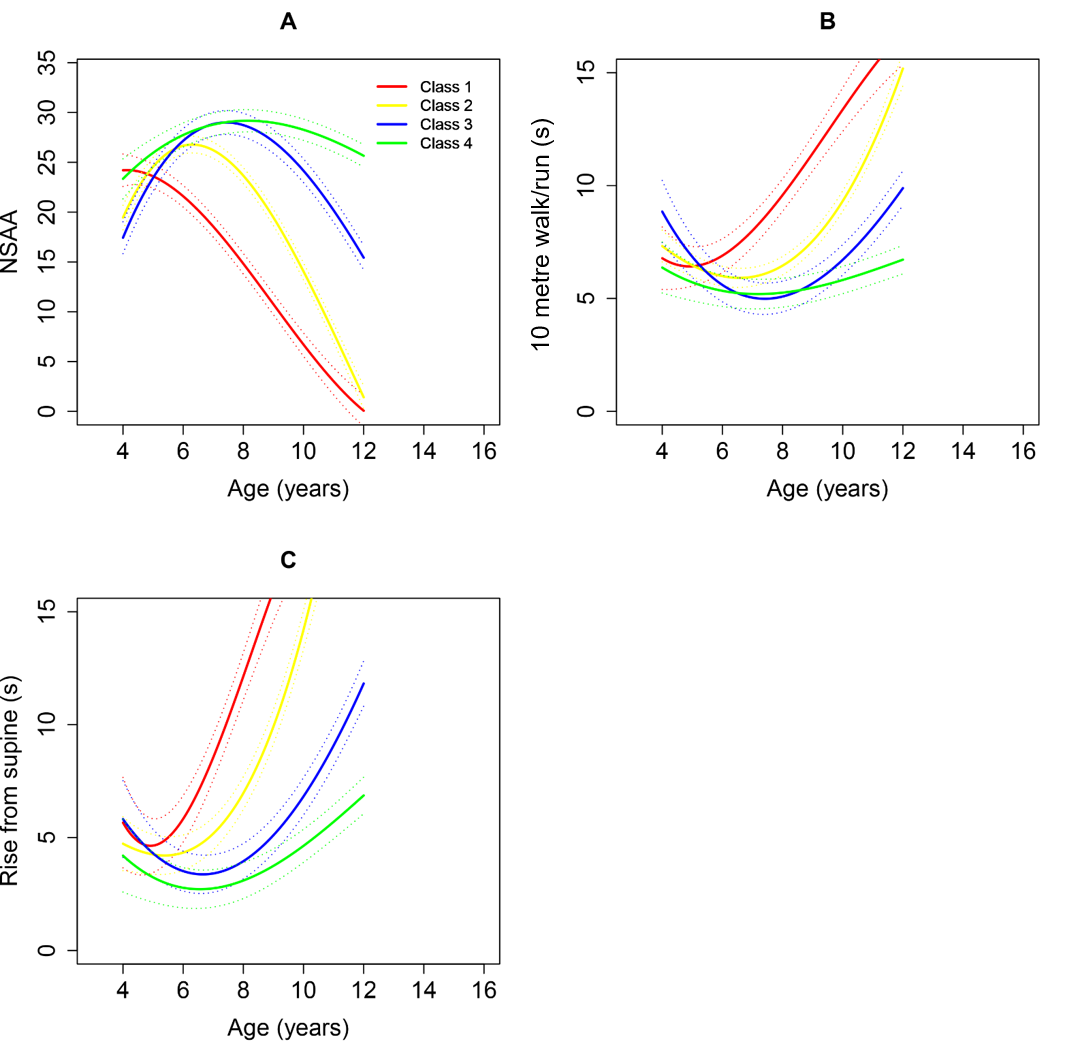

Supplement: S2 Fig — Fitted mean trajectories of (A) NSAA total score, (B) 10 metre walk/run completion time and (C) timed rise from supine, all stratified by latent class. NSAA, North Star Ambulatory Assessment; s, seconds. (TIF) [file pone.0221097.s003.tif]
